# Schizophrenia: Impact on quality of life

**DOI:** 10.4103/0019-5545.43632

**Published:** 2008

**Authors:** Ram Kumar Solanki, Paramjeet Singh, Aarti Midha, Karan Chugh

**Affiliations:** Department of Psychiatry, SMS Medical College, Jaipur, India; 1 Santokba Durlabh Ji Memorial Hospital, Jaipur, India; 2 GMC, Rajindera Hospital, Patiala, India

**Keywords:** Quality of life, schizophrenia, positive and negative syndrome scale

## Abstract

**Aims:**

The purpose of the present study was to assess quality of life (QOL) in patients with schizophrenia and to determine influence of clinical factors and socio-demographic variables on QOL of schizophrenic patients.

**Setting and Design:**

Cross-sectional study carried out on outdoor patients attending Department of Psychiatry, SMS Medical College, Jaipur, India.

**Materials and Methods:**

Fifty patients of schizophrenia diagnosed as per ICD - 10 with minimum duration of illness being two years and attending out patient department (OPD) at psychiatric centre or psychiatric clinic at SMS medical college, hospital, Jaipur, India for maintenance treatment fulfilling the criteria given below were registered. They were evaluated using positive and negative syndrome scale (PANSS) and Quality of Life Instrument (WHO QOL - BREF). The data collected on above tools, was analyzed using descriptive and inferential statistics using Pearson correlation coefficient.

**Results and Conclusions:**

Patients were having lowest QOL scores in social relationships domain of WHO QOL - BREF scale. Social relationship domain of QOL was significantly negatively correlated with occupation with employed patients reporting better QOL in this domain. There were significant positive correlation of total monthly income with social relationship domain and total QOL. There were no statistically significant correlation between QOL parameters and clinical characteristics in schizophrenics. Scores on positive subscale and total PANSS were significantly negatively correlated with physical, Psychological, social relationship domains and total QOL. Negative subscale had significant negative correlation with physical and psychological domains and total QOL. General psychopathology subscale had significant negative correlation with all subscales of QOL. This study confirms poor QOL in schizophrenia despite significant improvement with pharmacological treatment.

## INTRODUCTION

The concept of quality of life (QOL) has assumed special significance in the medical field in the wake of progressive movement towards rehumanizing ‘hi-tech’ medicine; QOL refers to subjective satisfaction experienced by an individual with regard to his or her physical, mental and social sphere. The concept of QOL is perhaps more important in those disorders which run a chronic and debilitating course and where the treatment is mostly of a noncurative nature and continues over a long period. Quality of life research in psychiatry is still in its infancy, although in recent years attempts have been made to generate measures of QOL.[1] Quality of life research faces many problems, including the lack of a universally accepted definition, lack of consensus about domains of QOL, lack of psychometrically valid instruments, subjective vs. objective assessment, generic vs. specific instruments, problems of assessment in multicultural, multiethnic societies and limitation of the influence of culture on the measurement of QOL, etc.[2]

Sullivan G and others[3] also noted that long term psychiatric disorders are more vulnerable to stress, are more dependent, have greater deficits in living skills and have greater problems in employment and in relationship to their social environment. They also reported that QOL of chronic psychiatric patients (heterogeneous groups including patients with schizophrenia, chronic affective disorders, personality disorders, substance abuse, etc.) is impoverished especially in the domains of housing conditions, family environment, social network, financial circumstances, safety and practical skills.

Schizophrenia is a severe and debilitating disorder, which affects general health, functioning, autonomy, subjective well being, and life satisfaction of those who suffer from it. Despite 50 years of pharmacological and psychosocial intervention, schizophrenia remains one of the top causes of disability in the world.[4]

WHO initiated a series of studies on QOL in relation to health and disease. There was a WHO multisite QOL study aimed at development of a generic questionnaire to measure QOL across different illness groups in Madras and Delhi. In Madras they focused on QOL in schizophrenic patients who have partially or totally recovered. Psychological, interpersonal and sociocultural factors that had impact on the life of schizophrenia patient had been assessed in a manner so as to act as guide for better management and coping with the illness in them. These strategies and interventions improved the functioning of the schizophrenia patients, reduced their disability due to illness, improved productivity and self esteem, prevent relapse and so on.[5] Most studies of QOL had been conducted in developed countries. In developing countries, differences in the course of mental disorders and influence of cultural factors on the progress of schizophrenia, had been documented by Kulhara.[6]

We undertook this study to assess and quantify QOL in patients suffering from schizophrenia attending Psychiatric Centre, Jaipur. This study has been carried out as an attempt to assess impact of schizophrenia on different domains of patient's life. This would help to understand and plan accordingly and appropriately as far as their management and rehabilitation is concerned.

### Aims and objectives

The general aim of the present study was to assess QOL in schizophrenia and also to determine factors influencing them. Keeping this in mind, following specific aims were formulated.

- To assess QOL in patients with schizophrenia.
- To determine influence of clinical factors and socio-demographic variables on QOL schizophrenic patients.

## MATERIALS AND METHODS

To fulfill the above aims and objectives the present study was undertaken.

### General design of the study

#### Sample of the study:

For the purpose of present study, initially 50 patients of schizophrenia diagnosed as per ICD - 10 with minimum duration of illness being two years and attending out patient department (OPD) at psychiatric centre or psychiatric clinic at SMS medical college, hospital, Jaipur, India for maintenance treatment fulfilling the criteria given below were registered.

### Inclusion criteria

- Patients fulfilling criteria for Schizophrenia as per ICD-10.
- Patients between 18-60 years of age.
- Patients of either sex.
- Patients were under maintenance treatment of atypical antipsychotic preferably risperidon.

### Exclusion criteria

- Illiterate patients
- Patient with primary diagnosis of depression and any comorbid psychiatric disorder.
- Patients with any chronic physical illness, organic brain disorder, or substance dependence.

A written consent of the patient and caregiver for participation in the study would be obtained. To be eligible as a caregiver for each patient, he/she must have a reasonable contact with the patient (at least two weeks) and over 16 years of age.[7]

Three patients dropped from schizophrenic group because they felt exhaustive during the interview and did not able to complete the proforma.

### Instruments of study

A specially designed proforma was used for evaluation of the patients. It included the following:

- Socio demographic data sheet.
- Clinical profile sheet.

#### Positive and negative syndrome scale (PANSS)

This 30-item, 7-point rating instrument was conceived as a carefully defined and operationalized method that evaluates positive, negative, or other symptom dimensions on the basis of a formal semi-structured clinical interview and other informational sources. In the 30 items, seven are grouped to form a positive scale, measuring symptoms that are superadded to a normal mental status, and seven items constitute negative scale, assessing features absent from a normal mental status, remaining 16 items constitute general psychopathology scale that gauges the overall severity of schizophrenic disorder by summation of remaining 16 items.[8]

#### Quality of Life Instrument (WHO QOL - BREF)

It is a structured self report interview it was developed by WHO division of mental health. It consists of 26 items. Its purpose is to assess QOL of person. It assesses patients under four domains which are physical, psychological, social and environmental. Its psychometric properties have been found to be comparable to that of full version WHO QOL -100.[9]

### Operational procedures

The subjects meeting the criteria laid down for the purpose of this study were explained all about the study and proper written consent was taken from patients and accompanying primary caregiver. Most patients were interviewed immediately after their registration at OPD, psychiatric centre, S.M.S. Medical College, Jaipur, during the period from May 2005 to April 2006. Those cases in which patient or caregiver were in hurry to return house or office and could not stay for interview, arrangements were made to interview them at an alternative time.

The interview was semi-structured and all information was recorded in a carefully designed structured proforma. Following this, the schizophrenic patients were administered PANSS and WHO QOL scale BREF version.

### Statistical analysis

Mean (SD) and percentages were used for descriptive purpose. To examine the relationship between independent variables (socio demographic and clinical) and dependent variable (QOL), Pearson's product moment correlation (if distribution was normal) and Spearman's rho (if distribution was not normal) was completed.

## RESULTS

The mean age of patients of schizophrenics groups was 36.43 years. Most of the respondents of our study were married, males, belonged to Hindu religion, joint families and urban background [Table 1]. Most of the patients had education above metric level. 30% of schizophrenic groups, respectively, were having total family monthly income of more than Rs. 6000. Table 2 shows clinical characteristics of subjects included in our study. The duration of illness of 72.3% of schizophrenic patients was more than five years. Most of the patients in our study groups had no past history or family history of psychiatric illness. Mean total score of PANSS is 57.09, which falls in the mild range of psychopathology [Table 3]. Patients were having lower QOL scores in social relationships domain of WHO QOL-BREF scale [Table 4]. Table 5 reveals that social relationship domain of QOL was significantly negatively correlated with occupation as employed patients reporting better QOL in this domain. There were significant positive correlation of total monthly income with social relationship domain and total QOL.

**Table 1:** Distribution of socio-demographic profile in schizophrenia groups

Variable	schizophrenia *n* = 47, f (%)
Age in (years)–mean (SD)	36.43 (10.65)
Sex	
Male	38 (80.9)
Female	9 (19.1)
Marital status	
Single	10 (21.3)
Married	36 (76.6)
Remarried	-
Widowed	-
Divorced	-
Separated	1 (2.1)
Occupation	
Professional/semi professional	5 (10.6)/1 (2.1)
Farmer	6 (12.8)
Skilled worker	1 (2.1)
Semi skilled worker/unskilled worker	9 (19.1)
Unemployed	14 (29.8)
Housewife	6 (12.8)
Retired	1 (2.1)
Student	4 (8.5)
Education	
Illiterate	-
Literate	-
Primary	1 (2.1)
Middle	10 (21.3)
Metric	14 (29.8)
Inter	12 (25.5)
Graduate	9 (19.1)
Post graduate	1 (2.1)
Monthly income	
Up to 1500	9 (18.0)
1501-3000	13 (26.0)
3001-6000	10 (26.0)
Above 6000	15 (30.0)
Religion
Hindu	43 (91.5)
Muslim	4 (8.5)
Sikh	-
Christian	-
Family type	
Nuclear	14 (29.8)
Joint/others	33 (70.2)
Locality	
Urban	32 (68.1)
Rural	15 (31.9)

**Table 2:** Distribution of clinical characteristics in schizophrenia groups

Variable	Schizophrenia (*n*=47) f (%)
Duration of illness	
2-5 years	13 (27.7)
>5 years	34 (72.3)
Past history of psychiatric illness	
Present	10 (21.3)
Absent	37 (78.7)
Family history of psychiatric illness	
Present	11 (23.4)
Absent	36 (76.6)

**Table 3:** Scores of positive, negative and general psychopathology subscales in schizophrenia group

Variable (PANSS)	Schizophrenia (*N*=47) mean (SD)
Positive	13.9 (5.02)
Negative	14.34 (6.09)
General psychopathology	29.55 (7.26)
Sums	57.09 (15.22)

**Table 4:** Quality of life scores in schizophrenia

Variable (WHO QOL BREF)	Schizophrenia (*n*=47) mean (SD)
Physical health	22.68 (5.72)
Psychological health	18.64 (5.43)
Social relationship	9.96 (2.83)
Environment	26.72 (5.30)
Total score	85.06 (17.72)

**Table 5:** Correlation between quality of life parameters (WHO QOL) and socio demographic characteristics in schizophrenic patients (*n*=47)

Variable	Physical health	Psychological health	Social relationship	Environment	Total score
Age in (years)	-.017	.075	-.017	-.123	-.015
Sex	.085	.123	-.186	-.150	-.013
Marital status	-.015	.194	-.013	-.123	.025
Occupation	-.225	-.107	-.309*	-.072	-.199
Education	.081	.206	.099	.286	.213
Monthly income	.232	.161	.307*	.271	.417**
Religion	.058	.120	.059	.132	.091
Family type	.095	.077	-.010	.285	.136
Locality	-.115	-.141	-.104	-.155	-.123

Pearson product moment (*r*), r sp Spearman rho.

* *P*<.05

** *P*<.01.

As shown in Table 6 there were no statistically significant correlation between QOL parameters and clinical characteristics in schizophrenics. Scores on positive subscale and total PANSS were significantly correlated in negative direction with physical, psychological, social relationship domains and total QOL. Negative subscale had significant negative correlation with physical and psychological domains and Total QOL. General psychopathology subscale had significant negative correlation with all subscales of QOL [Table 7].

**Table 6:** Correlation between quality of life parameters (WHO QOL) and clinical characteristics in schizophrenicpatients (*n*=47)

Variable	Physical health	Psychological health	Social relationship	Environment	Total score
Duration of illness	-.052	.011	-.094	-.085	-.002
Past history of psychiatric illness	-.167	-.142	-.287	-.285	-.237
Family history of psychiatric illness	.164	-.009	.279	.000	.138

Pearson product moment (*r*). *P<.05, ** P<.01.

**Table 7:** Correlation between quality of life parameters (WHO QOL) and severity of schizophrenic syndromes (*n*=47)

Variable (PANSS)	Physical health	Psychological health	Social relationship	Environment	Total score
Positive *r*	-.502**	-.341*	-.295*	-.216	-.403**
Negative *r*	-.517**	-.438**	-.205	-.101	-.398**
General psychopathology *r*	-.700**	-.621**	-.422**	-.329*	-.632**
Sums *r*	-710**	-.587**	-.382**	-.268	-.596**

Pearson product moment (*r*).

* P<.05

** P<.01.

## DISCUSSION

Estimates of unemployment in people with schizophrenia were 70-85%,[10] where as in our schizophrenic only 29.8% were found to be unemployed. This can be explained by the fact that in developed countries jobs are more complicated than in less advance societies.[11] It is also known that progress of schizophrenic patients is better in developing countries because of more handling of patients in families and in society and less institutionalization.[12]

Table 4 indicates the existence of impoverished QOL in schizophrenic patients. On QOL measures, schizophrenic patients had lowest scores on the social relationship domain. Patients of chronic mental illness dislike the stigma of mental illness, which excludes them from social life. These patients are subject to many different kinds of formal and informal discrimination.

In this study, lowest score on social relationship domain of QOL in schizophrenics could be due to the negative symptoms present in these patients, among which asociality, avolition and apathy are known to be prominent. This finding is supported by an earlier study by Gupta *et al.*[13] Occupation had a negative correlation with social relationship domain of QOL [Table 5] which means that employed patients attained a better QOL in social relationship domain. This could be explained by the fact that employed patients had aspirations to live like ‘normal people’ – having their own family, friends and other social relationships.

This findings is similar to earlier studies,[14–17] which demonstrated that many patients never married and were unemployed may reflect their deficits when interacting and coping with their human, social and physical environment and the complexity of modern society.

There were significant positive correlation of total family income and social relationship domain and total QOL [Table 5]. This is understandable as patients who were financially satisfied had expectations like normal people. Similar results about relationship of income and QOL were also reported by Cardoso SC *et al*.[18] Duration of illness, past history and family history of psychiatric illness had no significant correlation with QOL [Table 6].

Positive subscale and total score of PANSS were significantly correlated in negative direction with physical, psychological and social relationship domain and total QOL [Table 7]. Negative subscale had significant negative correlations with physical and psychological health domain and total QOL. General psychopathology subscale had significant negative correlation with all subscales of QOL and total QOL.

This co-relational analysis reported that all symptom clusters of PANSS are related to poor QOL. Among all, general psychopathology had high correlation with QOL. This finding is supported by various studies.

Packer *et al.*,[19] evaluated psychopathology in schizophrenia using brief psychiatric rating scale (BPRS) and found a high correlation between global QOL and BPRS total score as well as relationship between subjective QOL and negative and positive symptom clusters.

Galletly *et al*.[20] demonstrated that changes in QOL were most highly correlated with changes in general psychopathology. These correlations were higher than correlations between changes in QOL and changes in negative symptoms or changes in overall PANSS score.

Heslegrave *et al.*[21] also found a stronger relationship between general psychopathology scale of PANSS and subjective QOL than a number neuropsychological measures and subjective QOL. They reported that total PANSS scores and general psychopathology index were equally correlated with global QOL.

These results and the above studies imply that overall levels of general psychopathology are more highly associated with subjective QOL than are core positive and negative symptoms. General psychopathology contains items that include symptoms of depression and anxiety. The review of studies above suggested that anxiety and depression may be more critical than any other symptom of schizophrenia.

### Limitations and directions for future research

The results of the current study should be interpreted in the background of following limitations, which may have affected the observations:

- Current study, based exclusively on hospital based outpatient sample and therefore, is may not be the representative sample of patients in community.
- Patients with illness duration of two years or more were included to make the sample homogeneous. This, however, limits the generalization of results from the present study of schizophrenic respondents having acute illnesses.
- The QOL instrument WHO QOL-BREF used in current study is a generic instrument that was not designed specifically for schizophrenic patients, using a combination of both generic and specific instrument would have been the better choice. This instrument only assesses the subjective QOL, while the addition to objective measures might have been useful.
- All the variables were assessed cross-sectionally; hence answers to cause-effect relationship between variables cannot be given. Longitudinal studies should be carried out to look for correlations between changes in impact (variables) with changes in severity of illness, type of treatment, etc. to answer questions regarding causal connections.
- The sample size may be regarded as small and hence generalization of our findings to all types of patients is not possible.
- As chronic stable, co-operative patients are included in the study, data from more severe patients is missing.

Thus, future studies should be planned and carried out keeping the view in methodological limitations mentioned above.

## CONCLUSIONS

- This study confirms poor QOL in schizophrenia despite significant improvement with pharmacological treatment. Chronic stable patients showing impoverished QOL also require interventions that enhance QOL.
- The use of health related QOL questionnaires represents a step forward in the evaluation of treatment efficacy. These assessments can lead to better targeted interventions and more specific measures of response to treatment.
- This study also emphasize the need of refinement in QOL assessment and due consideration of its cultural aspects.

## References

[1] Malm U, Pra M, Dencker SJ (1981). Evaluation of quality of life of schizophrenic outpatients: A checklist. Schizophr Bull.

[2] Skantze K, Malm U, Dencker SJ, May PR (1990). Quality of life in schizophrenia. Nord J Psychiatry.

[3] Sullivan G, Wells KB, Leake B (1992). Clinical factors associated with better quality of life in a seriously mentally ill population. Hosp Commun Psychiatry.

[4] Murray CL, Lopez AD (1996). The global burden of disease.

[5] Kumar S, Sameul R, Prabhu R (1992). Schizophrenia and quality of life in relation to health and illness: Socio cultural issues at world congress of Social Psychiatry.

[6] Kulhara P (1994). Course and outcome of schizophrenia some transcultural observations with particular reference to developing countries. Eur Ach Psychiatr Clin Neurosci.

[7] Szmukles GI, Burgers P, Herman H (1996). Caring of relatives with serious mental illness - the development of experience of care giving inventory. Soc Psych Epidem.

[8] Kay SR, Fiszbun A, Opler LA (1987). The positive and negative syndrome scale (PANSS) for schizophrenia. Schizophr Bull.

[9] Saxena S, Chardiramani K, Bhargava R (1998). WHO QOL-Hindi A questionnaire for assessing quality of life in health care setting in India. Nat Med J India.

[10] Mulkern VM, Maderschied RW (1989). Characteristics of community program clients in 1980 and 1984. Hosp Comm Psychiatry.

[11] McGurk S, Meltzer HY (2000). The role of cognition in vocational functioning in schizophrenia. Schizophr Res.

[12] Ertugrul A, Ulug B (2002). The influence of neurocognitive deficits and symptoms on disability in Schizophrenia. Acta Psychiatr Scan.

[13] Gupta P, Kulhara P, Verma SK (1998). Quality of life in schizophrenia and dysthymia. Acta Psychiatr Scand.

[14] Rosenthal D (1970). Genetic theory and abnormal behaviour.

[15] Zubin J, Spring B (1977). Vulnerability a new view of schizophrenia. J Abnormal Psychol.

[16] Erikson R, Aberg R (1984). Valjard I Forandring: Levnadsvillkor, Sverege 1966-1981.

[17] Ciompi L, Straurs JS, Boker W, Brenner HD (1987). Toward a coherent multidimensional understanding and therapy of schizophrenia. Converging new concepts. Psychosocial treatment of Schizophrenia.

[18] Cardoso SC, Caiaffa TW, Bandeira M, Siqueira LA, Silva Abreu MN, Fonseca JO (2005). Factors associated with low quality of life in Schizophrenia. Ad Savde Publica Rio de Janerio.

[19] Packer S, Husted J, Cohen S, Tomlinson G (1997). Psychopathology and quality of life in schizophrenia. J Psychiatr Neurosoci.

[20] Galletly CA, Clark CR, McFarlane AC, Weber DL (1997). Relationships between changes in symptom ratings, neuropsychological test performance and quality of life in schizophrenic patients treated with clozapine. Psychiatr Res.

[21] Heslegrave RJ, Awad AG, Voruganti LN (1997). The influence neurocognitive deficits and symptoms on quality of life in schizophrenia. J Psychiatr Neurosoci.

